# Relating Vertex and Global Graph Entropy in Randomly Generated Graphs

**DOI:** 10.3390/e20070481

**Published:** 2018-06-21

**Authors:** Philip Tee, George Parisis, Luc Berthouze, Ian Wakeman

**Affiliations:** 1 Moogsoft Inc, San Francisco, CA 94111, USA; 2 School of Engineering and Informatics, University of Sussex, BN1 9RH Brighton, UK

**Keywords:** graph entropy, chromatic classes, random graphs

## Abstract

Combinatoric measures of entropy capture the complexity of a graph but rely upon the calculation of its independent sets, or collections of non-adjacent vertices. This decomposition of the vertex set is a known NP-Complete problem and for most real world graphs is an inaccessible calculation. Recent work by Dehmer et al. and Tee et al. identified a number of vertex level measures that do not suffer from this pathological computational complexity, but that can be shown to be effective at quantifying graph complexity. In this paper, we consider whether these local measures are fundamentally equivalent to global entropy measures. Specifically, we investigate the existence of a correlation between vertex level and global measures of entropy for a narrow subset of random graphs. We use the greedy algorithm approximation for calculating the chromatic information and therefore Körner entropy. We are able to demonstrate strong correlation for this subset of graphs and outline how this may arise theoretically.

## 1. Introduction and Background

### 1.1. Overview

Global measures of graph entropy, defined combinatorially, capture the complexity of a graph by specifically quantifying the information content of the graph as a function of the structure. In this context, complexity is a measure of how distinct or different each vertex is in terms of its interconnection to the rest of the graph. In many practical applications of network science, which can range from fault localization in computer networks to cancer genomics, this difference in connectivity can indicate that certain vertices in a graph are in some way more important to the correct functioning of the network the graph represents. In this paper, we explore the potential correlations between vertex level entropy measures [1,2] and global graph entropy [3]. This is an important problem for a very simple reason. Global graph entropy, as defined originally by Körner [3], is expensive to compute, as it relies upon the calculation of independent sets of the graph. This is a known NP-Complete problem, and in most real world graphs the computation of graph entropy is prohibitive. As stated before though, the value of this graph metric though is high, as it fundamentally captures a measure of the complexity of the graph that has a range of practical applications.

In previous work [4], we performed an extensive analysis of how local measures of entropy at the vertex level could be used as a tool in fault localization. The main result of that paper was that this “vertex entropy”, analyzed in a number of forms, was able to be used as a mechanism to eliminate noisy events from a communications network efficiently, by identifying important nodes in the network. Additionally, we were able to demonstrate that across a narrow set of representative graphs (star, paths, cycles, and perfect graphs), the vertex entropies and global entropies behaved similarly in terms of extrema. The more general relationship between local and global measures of information across an arbitrary graph has previously not been considered in our prior work. Indeed, it has never been established to what extent there is a meaningful correlation between these information measures. The existence and nature of such a correlation is the focus of this work and is the principal contribution of this paper.

If it were possible to approximate the value of graph entropy with a much more easily computable metric, it would be possible to use entropy in these and potentially many other applications. The fundamental barrier to computability is the fact that the entropy calculation depends upon combinatoric constructs across the whole graph. If instead, a metric were available that was intrinsically local, that is computable for each vertex in the graph with reference to only the local topology of the graph, it would then be possible to efficiently calculate a value for each vertex and then simply sum these values across the graph to obtain an upper bound for the entropy of the graph. The fact that it is an upper bound is possible to assert by simply appealing to the sub additivity property of graph entropy. That is, for any two graphs $G_{1}$ and $G_{2}$, the entropy of the union of the graphs obeys $H\left(G_{1}\cup G_{2}\right)\leq H\left(G_{1}\right)+H\left(G_{2}\right)$.

Local measures of entropy at the vertex level have been advanced in work by Dehmer et al. [1,2,5,6,7], and developed by many other authors including in recent work we published [4] exploring the utility of vertex entropies in the localization of faults on a computer network. The formalisms used by Dehmer et al. and in our previous work differ in the construction of the entropies, and how graphs are partitioned into local sets. A primary motivation for this difference was motivated by the practical application of these measures described in [4]. The relation between the vertex entropy formalisms introduced by Dehmer, and global graph entropies have been analyzed [8] and the central focus of this paper is to explore how closely our definitions of vertex entropy approximate global entropies for two classes of random graphs, the traditional Gilbert graph [9], and the Scale-Free graphs first advanced by Barabási [10]. We restrict our experimental investigation to simple connected graphs, which in the case of Scale Free graphs arise naturally, for the Gilbert graphs we chose to focus on the giant component (GC) of the generated graph. For statistical significance, this further restricts the choice of connection probability to a range above the critical threshold at which a GC emerges.

In this section, we present an overview of both global and vertex graph entropy, before discussing the experimental analysis in Section 2. The data analysis produces a strong correlation between vertex and global entropy, which we seek to explain in Section 3, by comparing the limits on the values of chromatic information (as a proxy for graph entropy) of random graphs and the expected values for vertex entropy when considering an ensemble of random graphs.

The main finding of this paper is that for random graphs the correlation is strong and that we can construct arguments based upon probabilistic reasoning to explain how vertex entropy and chromatic information can be related. We conclude in Section 4 and point to further directions in this research. If the strong correlation that we describe in this paper, subject to more rigorous theoretical results that are beyond the scope of this paper, emerges as a more fundamental relationship, this opens up the use of vertex level measures to frame entropic arguments for many dynamical processes on graphs, including network evolution. Such models of network evolution have been advanced by a number of authors including Peterson et al. [11] and ourselves [12].

Before presenting the experimental and theoretical analysis of the possible correlation between local node and global measures of entropy, in the rest of this section, we will briefly survey the necessary concepts. In the discussion that follows we will highlight the fundamental difference between the two ways of computing an entropy. At the heart of the discussion is the difference between global and local structure. Global graph entropy, and chromatic information are defined across the whole graph, and any change to the global topology of the graph can in principle produce very different values for the entropy. Local vertex measures, however, are only ever defined in reference to a restricted knowledge of the local subgraph of a given vertex. As there are many different ways to produce a globally different graph with similar local topology, a correlation between local and global measures is a significant new result.

### 1.2. Global Graph Entropy

The concept of the entropy of a graph has been widely studied ever since it was first proposed by Janos Körner in his 1973 paper on Fredman-Komlós bound [3]. The original definition rested upon a graph reconstruction based upon an alphabet of symbols, not all of which are distinguishable. The construction begins by identifying with each member of an alphabet of *n* possible signals $X\;=\;\{x_{1},x_{2},\ldots x_{n}\}$, with a probability of emission $P_{i},i\in 1,2,\ldots ,n$ in a given fixed time period. Using this basic construction the regular Shannon entropy [13] is defined in the familiar way:

(1) $$H\left(X\right)=-\sum_{i=1}^{n}P_{i}\log_{2}P_{i}.$$

In [3], and beautifully explained in [14], János Körner introduced the concept of the entropy of a graph in terms of a modified version of Shannon’s original argument. Considering the alphabet *X*, as defined above, imagine that not all of the signals are distinguishable. In the analysis going forward, we adopt the normal notation of a graph G(V,E) as the combination of a set of vertices *V*, and the set of edges *E* that exist between the vertices. Further, all of our analysis is restricted to simple graphs containing no self-edges (that is edges that connect a vertex to itself). A graph can be constructed by mapping to the vertex set *V* each of the signals in the alphabet, so that $v_{i}\in V$ equates to $x_{i}$ and naturally associated with each vertex is a probability of emission of a signal $P\left(v_{i}\right)=P_{i}$, which is a fixed property of each vertex. Now, each of the vertices are connected with an edge $e_{i,j}\in E$, if and only if the two signals $x_{i},x_{j}$ are distinguishable. The automorphism groups of this graph are naturally related to the information lost (and hence entropy gained), by certain signals not being distinguishable. To avoid the definition involving complex constructions using these automorphism groups, Mowshowitz et al. [15] recast the definition in terms of the mutual information between the independent sets of the graph. An independent set is a collection of vertices which are not adjacent (i.e., there are no edges between them) [16]. As there is no adjacency between members of an independent set, it is also a valid chromatic class, that is the vertices can be “colored” identically. The optimal chromatic decomposition of a graph then amounts to the division of the vertex set into the minimum number of independent sets, such that a vertex belongs to only one independent set, at which point the independent sets and chromatic classes are identical. These sets then constitute the optimal coloring of the graph and collection of chromatic classes. To establish the Mowshowitz definition of graph entropy, let us imagine a process whereby we randomly select a vertex from the graph, according to a probability distribution P(V) for each vertex, which as the process of selection is uniform will be identically $\frac{1}{n}$ for each vertex in a graph of size *n*. Each vertex will in turn be a member of an independent set $s_{i}\in S$ (*S* is chosen to represent the independent sets to avoid confusion with *I* the mutual information). The conditional probability P(V|S) is the probability of selecting a vertex when the independent set that it belongs to is known. These probabilities capture important information concerning the structure of the graph. Associated with P(V|S) is a measure of entropy H(V|S), or the uncertainty in the first occurrence of selecting a vertex when the independent set is known. Using these quantities we define structural entropy as follows:

**Definition** **1.** *The structural entropy of a graph G(V,E), over a probability distribution P(V), H(G,P), is defined as*

(2) H(G,P)=H(P)−H(V|S)

*where S is the set of independent sets of G, or equivalently the set of chromatic classes.*

Closely related to this definition of entropy is chromatic information. This is defined in terms of the colorings of the graphs that divide the graph into subsets of *V* where each vertex in *V* has the same color label. Each graph has an optimal minimum set of colorings that can be achieved, the number of such sets being referred to as the chromatic number of the graph χ. These subsets are called *Chromatic Classes* $C_{i}$, with the constraint that ${⋃}_{i}C_{i}=V$. Chromatic information is then naturally defined as

**Definition** **2.** *The chromatic information of a graph of n vertices is defined as*

(3) $$I_{c}\left(G\right)=\min_{\left\{C_{i}\right\}}\left[-\sum_{i}\frac{|C_{i}|}{n}\log_{2}\left(\frac{|C_{i}|}{n}\right)\right]$$

*where the minimization is over all possible collections of chromatic classes, or colorings, of the graph $C_{i}$.*

Crucially, the chromatic information is closely related to the second term in Equation (2), H(V|S), and if we assume that the probability distribution *P* is uniform, we can relate the two quantities through the following identity.

(4) $$H\left(G,P\right)=\log_{2}n-I_{c}\left(G\right).$$

This relationship is derived in the overview by Mowshowitz [15] and indicates that the entropy is determined by the chromatic decomposition of the graph when the vertex probability distribution is uniform. The maximum value of entropy can be seen to be when chromatic information is minimized [4], this minimum being obtained by a perfect graph. We will make use of this identity in Section 2 as chromatic information is much more readily calculable than entropy in standard network analysis packages. It is also common in this measure of information to drop the *P* in H(G,P), as we are assuming the probability is uniform.

### 1.3. Local Entropy Measures

The computational challenges in calculating global entropy measures stem from the calculation of the independent sets of a graph, which is a known NP-Hard problem. Recent work by Dehmer et al. [1,2] provided a framework for the definition of a form of graph entropy defined at the purely local level of a node. In essence, Dehmer introduces the concept of a *j*-Sphere, $S_{i}^{j}$, centered at the *i*th node. Dehmer’s original definition relied upon subsets of vertices of a fixed distance from a given vertex $v_{i}$, where distance $d(v_{i},v_{j})$ is the shortest distance between distinct vertices $v_{i}$ and $v_{j}$ (i.e., i≠j). For a node $v_{i}\in V$, we define, for j≥1, the “*j*-Sphere” centered on $v_{i}$ as

(5) $$S_{i}^{j}=\left\{v_{k}\in V|d\left(v_{i},v_{k}\right)=j\right\}.$$

On these *j*-Spheres, Dehmer defined certain probability-like measures, using metrics calculable on the nodes such as degree as a fraction of total degree of all nodes in the *j*-Sphere, from which entropies can be defined. This locality avoids the computationally challenging issues present in the global forms of entropy.

In recent work [4], the authors extended this definition to introduce some specific local measures for Vertex Entropy, that is the graph entropy of an individual node in the graph. The analysis that was followed was based upon the concept of locality introduced in Dehmer et al., using the concept of a *j*-Sphere. In this work we will expand upon that analysis, and, instead consider the vertices of a graph as part of an ensemble of vertices, and the graph itself in turn as part of an ensemble of graphs.

Returning to the fundamental definition of entropy, it is a measure of how incompletely constrained a system is microscopically, when certain macroscopic properties of the system are known. For example, if we have an ensemble of all possible simple, connected graphs of order *N*, G(N), (that is |V|=N), we potentially have a very large collection of graphs. Further, we could go on to prescribe a further property such as average node degree 〈k〉 for the whole ensemble, and ask what is the probability of randomly selecting a member of the ensemble $G_{i}\left(N\right)\in G\left(N\right)$, that shares a given value of this property 〈k〉, and denote that as $P(G_{i})$. Following the analysis in Newman et al. [17], we can then define the Gibbs entropy of the ensemble as

(6) $$S_{G(N)}=-\sum_{G_{i}\in G\left(N\right)}P\left(G_{i}\right)\log_{2}P\left(G_{i}\right),$$

which is maximized subject to the constraint, $\sum_{G_{i}\in G\left(N\right)}P\left(G_{i}\right)\langle k_{i}\rangle =\langle k\rangle$. This analysis allows us to go from the observed value 〈k〉 to the form of degree distribution and then on to other properties for the whole ensemble. In essence in our work, we take a different approach in two regards. Firstly, we restrict ourselves to the vertex level, where we consider that the vertices of an individual graph are themselves a randomized entity, which can be assembled in many ways to form the end graph. For example, if we were to decompose a given graph into the degree sequence of the nodes, there will be many nodes of the same degree, which does not completely prescribe which node in the graph we are considering. In that way, a measure of uncertainty and therefore entropy naturally arises.

The second difference to the approach taken by Newman et al. is that, rather than work from a measurable constraint and maximized form of entropy back to the vertex probability, we ask what probabilities we can prescribe on a vertex. In the interests of computational efficiency, we construct a purely local theory of the graph structure constrained to those vertex properties that are measurable in the immediate (that is j=1) neighborhood of the vertex. Out of the possible choices, we selected node degree, node degree as a fraction of the total edges in the network, and local clustering coefficient. In our work, we also redefined the local clustering coefficient $C_{1}^{i}$ of a node *i* as the fraction of existing edges amongst nodes in the j=1 neighborhood to possible edges in that neighborhood. This is different to the traditional measure in that it includes the edges between the node $v_{i}$ and its neighbors, a choice that was made to avoid a zero clustering coefficient for nodes that are the “center” of a star-like network. Following the analysis in our prior work [4], we summarize the considered probabilities below.

- **Inverse Degree:** In this case, we denote the vertex probability as
  (7) $$P(v_{i})=\frac{Z}{k_{i}^{\gamma }},\;\mathrm{with}$$
  (8) $$Z^{-1}=\sum_{j}k_{j}^{-\gamma }\;\mathrm{to}\;\mathrm{ensure}\;\mathrm{normalization}.$$
  This type of vertex probability mirrors the attachment probability of the scale-free model and leads to a power law of node degree. In the standard scale-free model γ=3, but for the purposes of our experimentation, and for simplicity, we set γ=1. In essence, very large hubs are less probable, which intuitively captures the notion that they carry more of the global structure, and therefore information, of the graph. Graphs comprised of nodes with similar degrees will maximize entropy using this measure, reflecting the fact that less information is carried by knowledge of the node degree.
- **Fractional Degree:** We use in this case the following for vertex probability:
  (9) $$P\left(v_{i}\right)=\frac{k_{i}}{2|E|}.$$
  This probability measure captures the likelihood that a given edge in the network terminates or originates at the vertex $v_{i}$. Nodes with a high value of this probability will be more highly connected in the graph, and graphs which have nodes with identical values of the probability will have a higher entropy. This reflects the fact that the more similar nodes are the less information is known about the configuration of a given node by simply knowing its fractional degree.
- **Clustering Coefficient:** The clustering coefficient measures the probability of an edge existing between the neighbors of a particular vertex. However, its use in the context of a vertex entropy needs to be adjusted by a normalization constant $Z=\sum_{i}C_{1}^{i}$ to be a well behaved probability measure and sum to unity. For simplicity, we omit this constant and assert the following:
  (10) $$P\left(v_{i}\right)=C_{1}^{i}.$$
  The local clustering coefficient captures the probability that any two neighbors of the node $v_{i}$ are connected. The larger this probability, the more the local one hop subgraph centered at $v_{i}$ is to the perfect graph. Again graphs comprised of nodes with similar clustering coefficient will maximize this entropy, reflecting the fact that the graph is less constrained by knowledge of the nodes clustering coefficients.

In essence for a given graph G(N)∈G(N), we specify a measured quantity for vertex $v_{i}$, as $x\left(v_{i}\right)=x_{i}$, and ask what is the probability of a random vertex $v_{i}$ having this value. We denote this probability as $P{\left(v_{i}\right)}_{x\left(v_{i}\right)=x_{i}}$. This allows us to define entropy at the vertex level, and for the whole graph as

(11) $$S(v_{i})=-P\left(v_{i}\right)\log_{2}P\left(v_{i}\right)$$

(12) $$S(G)=-\sum_{v_{i}\in G}P\left(v_{i}\right)\log_{2}P\left(v_{i}\right)$$

In our analysis, we compute the values of each of these variants of vertex entropy, summed across the whole graph as described in Equation (12). Because of the local nature of the probability measures, the vertex entropy values are far quicker to compute than any of the global variants and do not involve any known NP-Complete calculations.

### 1.4. Alternate Formulations of Entropy

Our definition of vertex entropy in Equation (12) follows the normal Shannon definition of entropy. This is not the most general form of an entropy, and is in fact a special case of the entropy formulations introduced by Alfred Rényi [18]. Indeed, for an alphabet of *n* signals $\left\{x_{i},x_{2},\ldots x_{n}\right\}$, each occurring with probability $p_{i}$ the normal properties of an entropy measure are satisfied by the more general expression:

(13) $$H_{\alpha }=\frac{1}{1-\alpha }\log_{2}\left(\sum_{i}^{n}p_{i}^{\alpha }\right),$$

which in the limit α→1 can be seen by application of L’Hôpital’s rule to yield the normal form for the Shannon entropy. The special case of α=2 is often termed the “Rényi entropy” or collision entropy and is so called because it measures the likelihood of two random variables drawn from the same distribution having the same value. For our purposes, the collision entropy is an interesting quantity to investigate, as it captures the likelihood of different vertices sharing the same local topology, according to the probability measures outlined above. In Section 2.4, we analyze the random graphs using a variant of our vertex entropies formulated using this collision entropy. This analysis concurs with our results using Shannon entropy.

## 2. Experimental Analysis

### 2.1. Method and Objectives

We seek to establish whether there is any strong correlation between the global graph entropy of a graph, and the entropy obtained by summing the local node values of entropy. Because of the computational limits involved in calculating global values of entropy, we are restricted to graphs of moderate size, which rules out analyzing repositories of real world graphs such as the Stanford Large Network Dataset [19], or the Index of Complex Networks (ICON) [20]. A more tractable source of graphs are randomly generated graphs, where we can control the scale.

Random graphs are well understood to replicate many of the features of real networks, including the “small world” property, clustering and degree distributions. We consider in our analysis two classes of randomly generated graphs: the Gilbert random graph G(n,p) [9] and the scale-free graphs generated with a preferential attachment model [10]. For both types of graphs, we simulated a large number of graphs with varying parameters to generate many possible examples of graphs that share a fixed number of vertices, with the choice of Gilbert graphs permitting varying edge counts and densities. For the purposes of our analysis, we fixed the vertex count at n=|V|=300, and in each case we included only fully connected, simple graphs. For the Gilbert graphs, this entailed analyzing only the giant component (GC) to ensure a fully connected graph.

We will discuss the results for each type of graph in more detail below, but the data revealed a strong correlation between the chromatic information of the graph (and therefore structural entropy), and the value obtained by summing the local vertex entropies. We considered three variants of the vertex entropy and included the edge density of the graph, defined as

(14) $$C\left(G\right)=\frac{2|E|}{n(n-1)}.$$

This measures the probability of an edge existing between any two randomly selected vertices.

To quantify the nature of the correlation between each of the aforementioned local entropies (including edge density) and chromatic information of the graphs, we adopted a model selection approach by performing polynomial regression using polynomials of increasing order up to 5, referred to as $H_{1,\ldots ,5}$ henceforth, using the technique of least squares to numerically calculate the polynomial coefficients. The best model (within the family of models considered) was assessed based on the Bayesian information criterion (BIC), and Akaike information criterion (AIC) [21].

To calculate the measures, we made the assumption that the distribution of the errors are identically and independently distributed, and reduced the likelihood function to the simpler expressions:

(15) $$BIC=n\log_{e}\left({\hat{\sigma }}_{r}^{2}\right)+k\log_{e}n$$

(16) $$AIC=n\log_{e}\left({\hat{\sigma }}_{r}^{2}\right)+2k$$

(17) $${\hat{\sigma }}_{r}^{2}=\frac{1}{n}\sum_{i=1}^{i=n}{\left({\hat{y}}_{i}-y_{i}\right)}^{2}$$

where ${\hat{y}}_{i}$ is the prediction by model *H*, ${\hat{\sigma }}_{r}^{2}$ the residual sum of squares, *k* the number of parameters in the model (in this case for $H_{j}$, k=j+1), and *n* the number of data points.

The graph generation, analysis, and model selection was all performed with a mixture of Java and MATLAB code, which is available upon request from the authors.

### 2.2. Scale-Free Graphs

**Figure 1:**
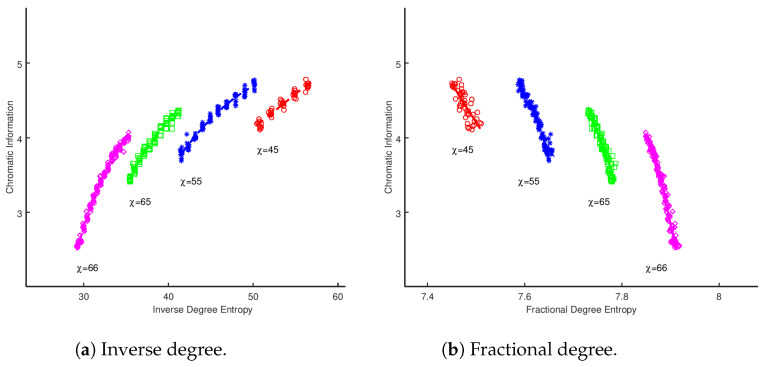

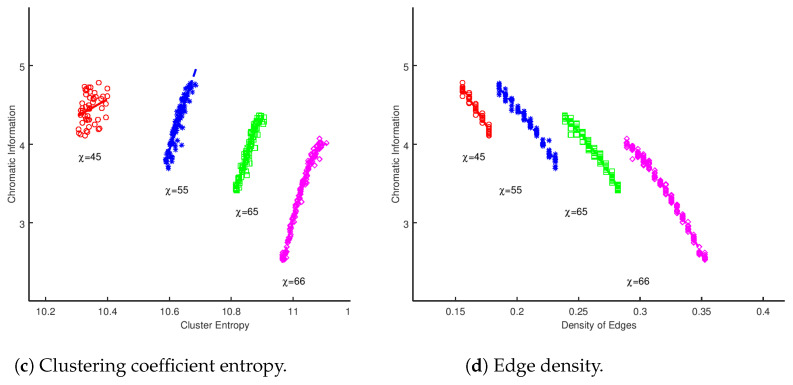
Sum of vertex entropies for whole graph vs. chromatic information for Barabási–Albert scale-free graphs of constant |V|.

The data for the scale-free graphs is displayed in Figure 1. We have segregated the plots by the calculated chromatic number for the graphs and overlaid the optimal least squares fit model. It is suggestive that, for graphs that share the same value of χ, there is a non-trivial, strong correlation between the metrics. To gain insights into the nature of this correlation, the data was fitted using a least squares approach to polynomials up to the 5th degree. In Table 1, Table 2, Table 3 and Table 4, we applied both the BIC and AIC to identify the best model. We have highlighted the row for the model with the strongest performance in the BIC test, and the $\Delta_{BIC}/\Delta_{AIC}$ is measured from the $H_{1}$ model, which performs worse in both analyses. In each case, there is strong support for the existence of a strong correlation between the vertex entropy measures and chromatic information. Both AIC and BIC have a marginal preference for higher order polynomial models, with rejection of higher order, indicating a preference for $H_{2}$ for inverse degree and edge density. The other measures require higher order fitting, over $H_{4}$, necessary for fractional degree and $H_{3}$ for cluster entropy.

### 2.3. Gilbert Random Graphs G(n,p)

**Figure 2:**
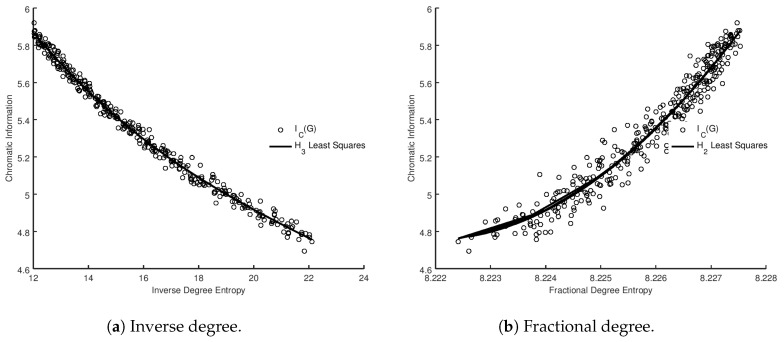

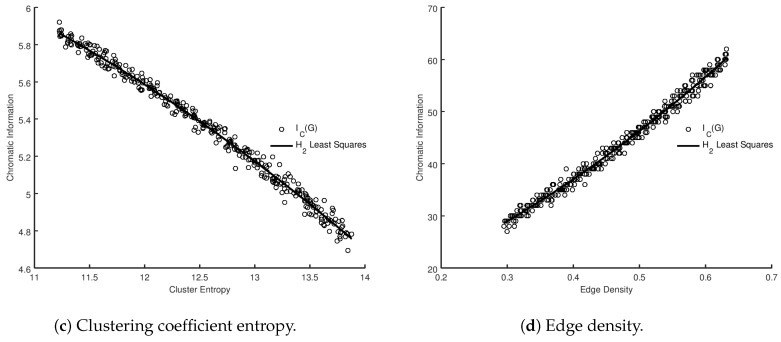
Sum of vertex entropies for whole graph vs. chromatic information for Gilbert graphs G(n,p) for p∈[0.31,0.7].

In addition to scale-free graphs we analyzed random graphs. The results are displayed in Figure 2, overlaid with the least squares optimized best fit. On visual inspection, it appears that there is a systematic and non-trivial correlation between the metrics. In order to gain insights into this correlation, the data was again fitted using a least squares approach to polynomials up to the 5th degree. In Table 5, Table 6, Table 7 and Table 8, we applied both the BIC and AIC to select the best model (among the models considered). As in the case of the scale-free graphs, we have highlighted the row in bold corresponding to the best model from a BIC perspective, and $\Delta_{BIC}/\Delta_{AIC}$ is measured against the worst performing model $H_{1}$. For Gilbert graphs, AIC and BIC both support the hypothesis of a strong correlation between the metrics. In the case of all but the inverse degree, it would appear that $H_{2}$ is an optimal choice of model. The inverse degree, however, would appear to be best fitted by $H_{3}$, in contrast to the behavior of the scale-free graphs.

### 2.4. Alternate Entropy Formulations

**Figure 3:**
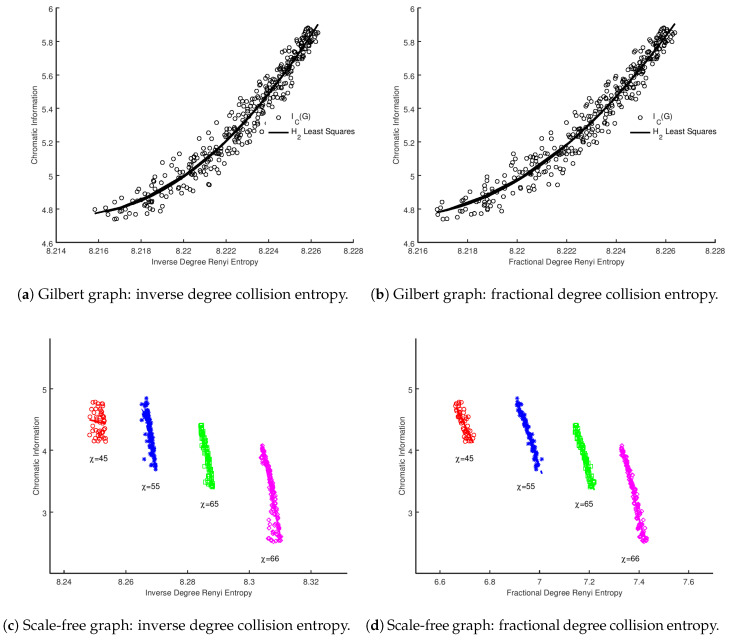
Sum of collision vertex entropies for whole graph vs. chromatic information for Gilbert graphs G(n,p) for p∈[0.31,0.7] and scale-free graphs of constant |V|.

Following on from the observation in Section 1.4, we performed our calculations using the same vertex probabilities, but converted into a collision entropy using Equation (13). We restricted our analysis to inverse degree and fractional degree for brevity, as the results are somewhat similar to the regular Shannon entropy variants of these measures. We present the results for both Gilbert and scale-free graphs in Figure 3. Similar to the results using Shannon entropy, visual inspection would indicate a strong correlation between the local and global measures, and this is borne out in the model selection analysis summarized in Table 9, Table 10, Table 11 and Table 12. The results of the model selection analysis are also broadly in line with the Shannon entropy case, favoring a quadratic and cubic relationship, with curiously inverse degree favoring a linear model. We conclude that the analysis supports that, even with an alternative formulation of entropy based upon vertex probabilities, there is a strong correlation between the local and global values.

## 3. Theoretical Discussion of the Results

The strong correlation between chromatic information and the various forms of vertex entropy derived graph entropies may at first seem paradoxical. The first quantity is combinatorial in nature and depends upon the precise arrangement of edges and vertices to produce the optimal coloring, which dictates its value. The summed vertex entropies, at least to first order, depend solely upon the individual node degrees and take little or no account of the global arrangement of the graph.

It is certainly beyond the scope of this work, and indeed to the opinion of the authors, intractable to calculate a precise relationship between the two quantities. It is possible, however, to construct an argument as to why the two quantities might be in such strong correlation. A number of approaches have been made to obtain upper and lower bounds for entropy measures in random and more general graphs [6,22] and in the analysis that follows, we will make use of a bounding approach to identify how the correlation between vertex entropy and chromatic information may arise.

To begin our analysis, it must first be noted that the experimental data is generated by sampling a number of randomly generated graphs of varying size. The only relationship between the graphs is the manner of their construction, and, crucially the resultant degree distributions of the graphs. Let us consider an ensemble of graphs G(G(V,E)), with degree distributions P(k) and fixed order n=|V|. For this ensemble, as we do not know in advance the structure of the chromatic decomposition of a given graph, we can calculate the chromatic information for an “averaged” member *G* of order *n*, and chromatic number χ(G), by using the average size of a chromatic class as follows:

(18) $${\bar{I}}_{C}\left(G\right)\approx -\chi \left(G\right)\times \frac{\langle |C_{\alpha }|\rangle }{n}\log_{2}\frac{\langle |C_{\alpha }|\rangle }{n}$$

where $\langle |C_{\alpha }|\rangle$ is the average size of the chromatic class. This quantity, although not an actual expectation value, can be used to provide an upper bound on chromatic information (and conversely the minimum of entropy) as discussed below. In order to establish that this expression acts as an upper bound, it is sufficient to note that $\sum_{\alpha }\frac{|C_{\alpha }|}{n}=1$, and Equation (3) must be maximized subject to this constraint. It is a standard result that this is satisfied when $|C_{\alpha }|$ are identical for all chromatic classes. Equation (18) simply sets them to be an identical partitioning of vertices by chromatic number.

Using the definitions of vertex entropy described in Section 1, we can similarly compute an average value of each vertex entropy for a member of the ensemble G, where we have taken the continuum approximation in the integral on the right hand side:

(19) $${\bar{S}}_{vertex}=n\times \langle S\left(v_{i}\right)\rangle =n\int_{1}^{\infty }P\left(k\right)S\left(k\right)dk.$$

In the case of the scale-free graphs, this yields analytically soluble integrals for inverse degree, fractional degree vertex entropies, and edge densities but for the clustering coefficient entropies, and for all G(n,p) random graphs the integrals are not solvable directly. To simplify the analysis, we use the approximate continuum result for the scale-free degree distributions at t→∞, $P\left(k\right)=\frac{2m^{2}}{k^{3}}$, where *m* is the number of nodes a new nodes connects to during attachment. For the clustering coefficient, we can make a very rough approximation in the case of scale-free networks of 4m/n by arguing that, for an average node degree of 〈k〉=2m, each of the neighbors shares the average degree and has a probability if 2m/n of connecting to another neighbor of the vertex. The quantity is not exactly calculable, but in [10], a closer approximation gives $C_{i}^{1}\propto n^{-3/4}$, though for simplicity we will use our rough approximation. Where an exact solution is not available, we can roughly approximate the value of $\langle S\left(v_{i}\right)\rangle$, by replacing the exact degree of the node by the average degree and then asserting

(20) $$\langle S_{vertex}\rangle =n\times S\left(\langle k\rangle \right).$$

We summarize these expressions in Table 13.

In Section 2, we presented the analysis of samples of randomly generated graphs created using three schemes. Each of these showed a surprisingly strong correlation between the vertex entropy measures, summed across the whole graph, and the chromatic information obtained using the greedy algorithm. The greedy algorithm is well known to obtain a coloring of an arbitrary graph which is close, but not optimal. Indeed, the chromatic number of the graph obtained from the greedy algorithm $\chi_{g}\left(G\right)$ is an upper bound of the true chromatic number χ(G). For a full description, see [9,16].

### 3.1. Gilbert Random Graphs

Let us first consider the case of the Gilbert random graphs. We follow the same treatment and notation as in [9,23]. We construct the graph starting with *n* vertices, and each of the $\frac{1}{2}n\left(n-1\right)$ possible links are connected with a probability *p*. The two parameters *n* and *p* completely describe the parameters of the generated graph graph, and we denote this family of graphs as G(n,p). It is well known that Equation (18) is maximized when each of the chromatic classes of the graph $C_{\alpha }$ are uniform. That is, if the cardinality of a chromatic class is denoted by $\left|C_{\alpha }\right|$, and χ is the chromatic number of the graph, we have

(21) $$\left|C_{\alpha }\right|=\frac{n}{\chi },\;\forall C_{\alpha }.$$

This chromatic decomposition is only obtained from the perfect graph on *n* vertices, $K_{n}$, and proof of this upper bound is outlined in [4]. For a given random graph G(n,p), we denote the coloring obtained in this way, the homogenized coloring ${\bar{C}}_{\alpha }$ of G(n,p), and we assert that $I_{C}\left(G\right)\leq {\bar{I}}_{C}\left(G\right)$. It is easy to verify that this yields the following as an expression for the chromatic information:

(22) $$I_{C}\left(G\right)\leq {\bar{I}}_{C}\left(G\right)=\log\chi .$$

To build upon the analysis, we consider a randomly selected chromatic class $C_{\alpha }$, which has $c=\frac{n}{\chi }$ nodes. For brevity of notation, we write c(χ) as *c* and ask the reader to remember that *c* is a function of the chromatic number of the graph. We denote the probability that *c* randomly selected nodes do not possess a link between them as $\bar{P}\left(C_{\alpha },\chi \right)$, and the probability that *at least* one link exists between these nodes as $P(C_{\alpha },\chi )$. We consider a large ensemble of random graphs, generated with the same Gilbert graph parameters n,p, which we write as G(n,p). For a randomly chosen member of this ensemble, the criterion for the graph G(n,p)∈G(n,p) to possess a chromatic number χ is simply that it is more likely for *c* randomly selected nodes in G(n,p) to be disconnected. That is,

(23) $$\bar{P}\left(C_{\alpha },\chi \right)\geq P\left(C_{\alpha },\chi \right).$$

To estimate the first term, we note that for *c* randomly chosen nodes to have no connecting edges is given by

(24) $$\bar{P}\left(C_{\alpha },\chi \right)={\left(1-p\right)}^{\frac{1}{2}c\left(c-1\right)}.$$

We can also estimate the second term, by factoring the probability of a link from any of the nodes $v_{i}\in C_{\alpha }$, connecting to another node in $C_{\alpha }$. The total probability is accordingly the product of

$$P\left(C_{\alpha },\chi \right)=P\left(\mathrm{of}\;\mathrm{any}\;\mathrm{link}\right)\times P\left(\mathrm{link}\;\mathrm{connects}\;\mathrm{two}\;\mathrm{nodes}\;v_{i},v_{j}\in C_{\alpha }\right)\times c.$$

Feeding in the standard parameters from the Gilbert graphs, we obtain

$$\begin{array}{cc} P(C_{\alpha },\chi ) & =p\times \frac{c(c-1)}{n(n-1)}\times c\\ & =\frac{pc^{2}\left(c-1\right)}{n(n-1)}. \end{array}$$

If we substitute back in the dependence of *c* upon the chromatic number, we obtain the following inequality, as the criteria for a given chromatic number χ to support an effective random coloring of the graph, that can be used to estimate χ:

(25) $${\left(1-p\right)}^{\frac{1}{2}c\left(c-1\right)}\geq \frac{pc^{2}\left(c-1\right)}{n(n-1)}.$$

**Figure 4:**
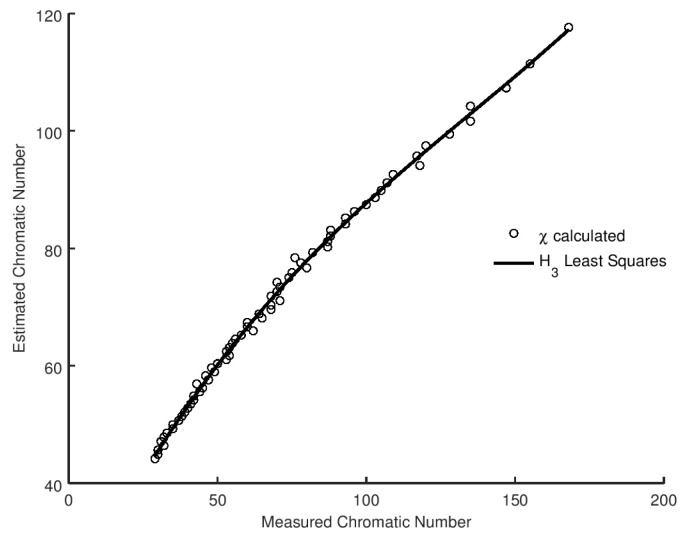
Calculated χ versus measured χ, for Gilbert graphs G(n,p) with n=300 and p∈[0.3,1.0]. Overlaid is the least squares fit for $H_{3}$.

It is easy to verify that equality is only ever reached when p→1, which yields the perfect graph in which c=1, as every node is adjacent to every other node. The left hand side of Equation (25) is an increasing function of χ, whereas the right hand side is a decreasing function, so as χ increases we arrive at a minimum value such that the inequality is satisfied. We take this to be our estimate of the value of χ. The obtained value is not a strict upper bound, as we see in Section 2, but it is a reasonable estimate of the value. Although Equation (25) as a transcendental equation is not directly soluble, we can numerically solve to determine the value of χ for a fixed link probability *p* at which equality is reached. We present the results of the analysis in Figure 4, together with the optimal least squares fit of the relationship. We can also evaluate the best model for the correlation between our estimate and the measured values using BIC and AIC in the same manner as with the metrics. We present the results in Table 14. It is evident that a cubic relationship offers the best choice of model, and that fit is overlaid on the experimental data in Figure 4.

Having established that we can use Equation (25) to generate a good estimate of the chromatic number of a Gilbert graph, we can now attempt to explain how the chromatic information obtained from the greedy algorithm correlates with the vertex entropy measures. We begin by simplifying Equation (25) to determine the minimum value of χ at which equality is reached, by assuming the limit of c≪n, and c,n≫1 to obtain

$${\left(1-p\right)}^{\frac{n^{2}}{2\chi^{2}}}=\frac{pn}{\chi^{3}}.$$

Taking the logarithm of both sides of this equation and manipulating we arrive at the following expression:

(26) $$3\log\chi =\log pn-\frac{n^{2}}{2\chi^{2}}\log\left(1-p\right)$$

(27) $${\bar{I}}_{C}\left(G\right)=\frac{1}{3}\log pn-\frac{n^{2}}{6\chi^{2}}\log\left(1-p\right).$$

Equation (27) represents an approximation for the chromatic information of a random graph. Numerical experimentation indicates that the first term dominates for small values of *p*, and as p→1.0 the second terms becomes numerically larger. Inspection of Table 13 shows that Equation (27) contains terms that reflect a number of the expressions for the vertex entropy quantities we have considered. Indeed, as the experiments were conducted at a fixed value of n=300, for small *p*, by elementary manipulation, one can see that ${\bar{I}}_{C}G\propto pS_{VE}$, or alternatively ${\bar{I}}_{C}G\propto S_{CE}/p$. Although this is a far from rigorous analytical derivation of the dependence of the chromatic information on the vertex entropy terms, the analysis does perhaps go some way to making the strong correlation experimentally make sense in the context of this theoretical analysis. Deriving an exact relationship between the two quantities is beyond the scope of this work.

### 3.2. Scale-Free Graphs

In the case of scale-free graphs we can follow a similar analysis to that of the Gilbert graph models. To derive the probability of a link, we can appeal to the preferential attachment model. For a randomly selected node, the average probability of acquiring a link *p* is computable as p=〈k〉/2mt. The dependence upon the connection valence *m* drops out and the average probability of a link existing between two nodes becomes p=1/n.

Following an identical argument to the random graph case, we arrive at the following relationship:

(28) $${\bar{I}}_{C}G=\frac{n^{2}}{4\chi^{2}}\log_{2}\left(\frac{n}{n-1}\right).$$

Again, it is important to stress that this is not a rigorous derivation of a relationship between chromatic information and vertex entropy, but it is possible to explain some of the correlations in the results presented in Figure 1. For a given fixed value of χ, the experiments represent graphs produced with increasing edge densities. As edges are added to the graph that *do not* increase the chromatic number, the size of the chromatic classes will evidently equalize (a discussion of this point can be found in [4]). This will have the effect of increasing the chromatic information, until a point is reached where χ→χ+1. At this point, the denominator of Equation (28) will increase, causing a drop in chromatic information. Therefore, as each vertex entropy measure is fundamentally dependent upon the number of edges in a fixed sized graph, we would expect to see a series of correlations for each value of chromatic information, which is indeed what is demonstrated in Figure 1.

## 4. Conclusions

In this work, we have principally been interested in investigating what, if any, correlation exists between purely local measures of graph entropy and global ones. It is not possible to make a general statement that, for any graph, this correlation exists, but for the two classes of random graphs considered, it is persuasive that a such a strong correlation exists.

This is an interesting and important result.

It is interesting to speculate what the connection could be between local measures of entropy at the vertex level and global measures, and indeed whether the two quantities are both measuring information. Perhaps the original definition of graph entropy provides a hint at how the two may be connected. In the original formulation, an edge exists between two vertices if the signals in the alphabet represented by the connected vertices are indistinguishable. The inability for the signals to be distinguished is a loss of information, and hence a gain in entropy, and the origin of the entropy of the graph follows. This loss of information is intrinsically local, so it is perhaps unsurprising that the vertex level measures, based upon degree, correlate to this. Of course, the local connectivity does not capture the entire subtle construction of the graph, which is what is captured by the global measures of entropy and why the local measures do not correlate exactly. The surprising part of our analysis was how well correlated local measures are for random graphs. This may well ultimately be a consequence of the nature of the construction of the graphs rather than a general result for all possible graphs.

There are other alternative measures of local connectivity that we could also have considered in our analysis; in particular, we have not considered spectral measures such as the Estrada index [24,25], which provide additional information on the degree to which a node participates in the subgraphs of a graph. It is beyond the scope of this work to extend the measures beyond those considered, but it would be an interesting avenue of further research to extend the investigation to a wider class of local properties of a graph.

Interest in the informational content of graphs has a wide range of application, both in terms of network dynamics as a model of network evolution. It is also under investigation outside the field of network science, in many different fields. In addition to dynamical models of network evolution, our formulation of vertex entropy has potential implications for dynamic processes on a network. For example, Ritchie et al. [26] demonstrated that describing a network in terms of global network metrics such as degree distribution and global clustering coefficient could obscure differences in local clustering with substantial impact on dynamics unfolding on that network. An intriguing possibility would therefore be that local entropy measures could encompass this higher-order structure and provide insights into dynamic processes on a graph, such as epidemic spread, and presents a further way to test the applicability of vertex entropy.

Beyond dynamic processes, it is possible that vertex entropy could also have relevance in the study of network resilience, in particular the vulnerability of real world networks to attack. Our work is based upon the Dehmer *j*-Sphere decomposition, and similar approaches have been used, for example, by Shang [27]. In further work, it would be interesting to investigate how our approach could be used to determine network resilience.

It is interesting to speculate what processes could underly the strong correlation between the local and global entropy measures. One possibility is that the random graphs produce sparse, tree-like graphs, which would cause the local entropy of the nodes to become solely dependent upon their degree. There has been work on the likelihood of generating such sparse graphs by Shang [28], but for the data sets that we generated there is more complex topology, including non-trivial clustering. In further work, we would like to investigate the interplay between entropy and sparsity.

The starting point for our theoretical investigation, Equation (18), is asserted as a relationship between the average chromatic class and the chromatic information. That assertion approximates the expectation value of the chromatic information if the size of the chromatic classes is closely bunched around a mean of narrow distribution. There have been recent contributions [29,30] that could potentially provide a mechanism to test how well the actual distribution of the cardinality of the chromatic classes is distributed according to a narrow Gaussian. Although beyond the scope of this paper, we view this as an interesting additional direction to take our research.

Outside network science, quantum gravity fundamentally relies upon spacetime becoming graph-like at the so-called Planck length [31]. With entropy becoming posited as a potential origin of gravity [32], the entropy of the spacetime graph is of interest. It would be convenient if large-scale information content was largely driven by the local graph structure of a typical node if a tractable entropic theory of quantum gravity is to become possible. If the entropy of the spacetime mesh were not definable locally, any theory obtained would suffer from a pathological lack of locality, a key feature of most modern field theories.

Although far from settled, this paper does at least illustrate that, for certain types of graph, the local environment of a typical node may indeed be a proxy for the information content of the graph. In future work, we intend to investigate other implications of vertex entropy on the dynamical processes possible on a graph.

## Figures and Tables

**Table 1:** Model selection analysis for inverse degree entropy for scale-free graphs of constant |V|.

Model	Bayesian Information Criteria	$\Delta_{\mathrm{BIC}}$	Akaike Information Criteria	$\Delta_{\mathrm{AIC}}$
$H_{1}$	−734.33	0.00	−740.33	0.00
$H_{2}$	−830.12	−95.80	−839.14	−98.80
$H_{3}$	−825.16	−90.84	−837.18	−96.85
$H_{4}$	−821.95	−87.62	−836.97	−96.63
$H_{5}$	−818.63	−84.31	−836.66	−96.32

**Table 2:** Model selection analysis for fractional degree entropy for scale-free graphs of constant |V|.

Model	Bayesian Information Criteria	$\Delta_{\mathrm{BIC}}$	Akaike Information Criteria	$\Delta_{\mathrm{AIC}}$
$H_{1}$	−708.84	0.00	−714.84	0.00
$H_{2}$	−715.24	−6.41	−724.25	−9.41
$H_{3}$	−719.49	−10.66	−731.51	−16.66
$H_{4}$	−719.62	−10.79	−734.64	−19.80
$H_{5}$	−715.31	−6.47	−733.33	−18.49

**Table 3:** Model selection analysis for cluster entropy for scale-free graphs of constant |V|.

Model	Bayesian Information Criteria	$\Delta_{\mathrm{BIC}}$	Akaike Information Criteria	$\Delta_{\mathrm{AIC}}$
$H_{1}$	−728.34	0.00	−734.35	0.00
$H_{2}$	−796.47	−68.12	−805.48	−71.13
$H_{3}$	−798.84	−70.50	−810.86	−76.51
$H_{4}$	−794.89	−66.54	−809.90	−75.55
$H_{5}$	−793.77	−65.43	−811.80	−77.44

**Table 4:** Model selection analysis for edge density for scale-free graphs of constant |V|.

Model	Bayesian Information Criteria	$\Delta_{\mathrm{BIC}}$	Akaike Information Criteria	$\Delta_{\mathrm{AIC}}$
$H_{1}$	−777.77	0.00	−783.78	0.00
$H_{2}$	−844.94	−67.17	−853.96	−70.18
$H_{3}$	−842.39	−64.62	−854.40	−70.63
$H_{4}$	−839.21	−61.43	−854.23	−70.45
$H_{5}$	−836.87	−59.10	−854.89	−71.11

**Table 5:** Model selection analysis for inverse degree entropy for random graphs of constant |V|.

Model	Bayesian Information Criteria	$\Delta_{\mathrm{BIC}}$	Akaike Information Criteria	$\Delta_{\mathrm{AIC}}$
$H_{1}$	−2004.92	0.00	−2008.75	0.00
$H_{2}$	−2181.68	−176.76	−2189.33	−180.59
$H_{3}$	−2182.62	−177.70	−2194.10	−185.35
$H_{4}$	−2176.82	−171.90	−2192.12	−183.37
$H_{5}$	−2171.14	−166.22	−2190.27	−181.52

**Table 6:** Model selection analysis for fractional degree entropy for random graphs of constant |V|.

Model	Bayesian Information Criteria	$\Delta_{\mathrm{BIC}}$	Akaike Information Criteria	$\Delta_{\mathrm{AIC}}$
$H_{1}$	−1806.14	0.00	−1809.96	0.00
$H_{2}$	−1874.29	−68.15	−1881.94	−71.98
$H_{3}$	−1868.70	−62.56	−1880.17	−70.21
$H_{4}$	−1859.34	−53.20	−1874.64	−64.68
$H_{5}$	−1856.25	−50.11	−1875.38	−65.42

**Table 7:** Model selection analysis for cluster entropy for random graphs of constant |V|.

Model	Bayesian Information Criteria	$\Delta_{\mathrm{BIC}}$	Akaike Information Criteria	$\Delta_{\mathrm{AIC}}$
$H_{1}$	−2109.61	0.00	−2113.44	0.00
$H_{2}$	−2146.19	−36.58	−2153.84	−40.40
$H_{3}$	−2140.43	−30.82	−2151.91	−38.47
$H_{4}$	−2134.61	−25.00	−2149.92	−36.48
$H_{5}$	−2128.86	−19.25	−2147.99	−34.56

**Table 8:** Model selection analysis for edge density for random graphs of constant |V|.

Model	Bayesian Information Criteria	$\Delta_{\mathrm{BIC}}$	Akaike Information Criteria	$\Delta_{\mathrm{AIC}}$
$H_{1}$	−951.67	0.00	−955.49	0.00
$H_{2}$	−985.93	−34.26	−993.58	−38.08
$H_{3}$	−980.15	−28.48	−991.63	−36.13
$H_{4}$	−974.37	−22.71	−989.68	−34.18
$H_{5}$	−969.80	−18.13	−988.93	−33.43

**Table 9:** Model selection analysis for inverse degree Renyi entropy for random graphs of constant |V|.

Model	Bayesian Information Criteria	$\Delta_{\mathrm{BIC}}$	Akaike Information Criteria	$\Delta_{\mathrm{AIC}}$
$H_{1}$	−1801.25	0.00	−1805.08	0.00
$H_{2}$	−1886.55	−85.30	−1894.20	−89.12
$H_{3}$	−1880.76	−79.51	−1892.23	−87.16
$H_{4}$	−1875.15	−73.90	−1890.46	−85.38
$H_{5}$	−1869.02	−67.77	−1888.15	−83.08

**Table 10:** Model selection analysis for fractional degree Renyi entropy for random graphs of constant |V|.

Model	Bayesian Information Criteria	$\Delta_{\mathrm{BIC}}$	Akaike Information Criteria	$\Delta_{\mathrm{AIC}}$
$H_{1}$	−1810.08	0.00	−1813.90	0.00
$H_{2}$	−1891.42	−81.34	−1899.07	−85.17
$H_{3}$	−1885.63	−75.56	−1897.11	−83.21
$H_{4}$	−1880.33	−70.25	−1895.63	−81.73
$H_{5}$	−1873.93	−63.86	−1893.06	−79.16

**Table 11:** Model selection analysis for inverse degree Renyi entropy for scale-free graphs of constant |V|.

Model	Bayesian Information Criteria	$\Delta_{\mathrm{BIC}}$	Akaike Information Criteria	$\Delta_{\mathrm{AIC}}$
$H_{1}$	−574.07	**0.00**	−580.08	**0.00**
$H_{2}$	−569.17	4.90	−578.18	1.90
$H_{3}$	−564.48	9.59	−576.50	3.58
$H_{4}$	−559.90	14.17	−574.92	5.16
$H_{5}$	−555.14	18.93	−573.16	6.92

**Table 12:** Model selection analysis for fractional degree Renyi entropy for scale-free graphs of constant |V|.

Model	Bayesian Information Criteria	$\Delta_{\mathrm{BIC}}$	Akaike Information Criteria	$\Delta_{\mathrm{AIC}}$
$H_{1}$	−694.87	0.00	−700.88	0.00
$H_{2}$	−690.61	4.25	−699.63	1.25
$H_{3}$	−698.94	−4.07	−710.96	−10.08
$H_{4}$	−695.68	−0.82	−710.70	−9.83
$H_{5}$	−690.75	4.11	−708.78	−7.90

**Table 13:** Average entropies across random graphs.

Vertex Entropy Measure	Scale-Free Graphs	Random Graphs *G*(*n*, *p*)
Inverse Degree	$2m^{2}n/9\ln2$	$p^{-1}\log_{2}\left(pn\right)$
Fractional Degree	$m\log_{2}\left(2mn\right)$	$\log_{2}n$
Clustering Coefficient	$4m\log_{2}\left(n/4m\right)$	$-np\log_{2}p$

**Table 14:** Model selection analysis for computed χ versus measured for |V|=300.

Model	Bayesian Information Criteria	$\Delta_{\mathrm{BIC}}$	Akaike Information Criteria	$\Delta_{\mathrm{AIC}}$
$H_{1}$	−90.00	0.00	−92.25	0.00
$H_{2}$	−138.79	−48.79	−143.28	−51.04
$H_{3}$	−146.53	−56.53	−153.28	−61.03
$H_{4}$	−142.72	−52.72	−151.71	−59.47
$H_{5}$	−138.60	−48.60	−149.84	−57.59

## References

[1] Dehmer M. (2008). Information processing in complex networks: Graph entropy and information functionals. Appl. Math. Comput..

[2] Dehmer M., Mowshowitz A. (2011). A history of graph entropy measures. Inf. Sci..

[3] Körner J. (1986). Fredman–Komlós bounds and information theory. SIAM J. Algebraic Discret. Methods.

[4] Tee P., Parisis G., Wakeman I. (2017). Vertex Entropy As a Critical Node Measure in Network Monitoring. IEEE Trans. Netw. Serv. Manag..

[5] Mowshowitz A., Dehmer M. (2012). Entropy and the complexity of graphs revisited. Entropy.

[6] Cao S., Dehmer M., Shi Y. (2014). Extremality of degree-based graph entropies. Inf. Sci..

[7] Emmert-Streib F., Dehmer M., Shi Y. (2016). Fifty years of graph matching, network alignment and network comparison. Inf. Sci..

[8] Dehmer M., Mowshowitz A., Emmert-Streib F. (2011). Connections between classical and parametric network entropies. PLoS ONE.

[9] Bollobás B. (2001). Random Graphs.

[10] Albert R., Barabási A.L. (2002). Statistical mechanics of complex networks. Rev. Mod. Phys..

[11] Peterson J., Dixit P.D., Dill K.A. (2013). A maximum entropy framework for nonexponential distributions. Proc. Natl. Acad. Sci. USA.

[12] Tee P., Wakeman I., Parisis G., Dawes J., Kiss I.Z. (2016). Constraints and Entropy in a Model of Network Evolution. arXiv.

[13] Shannon C.E. (1948). A Mathematical Theory of Communication. Bell Syst. Tech. J..

[14] Simonyi G. (1995). Graph entropy: A survey. Comb. Optim..

[15] Mowshowitz A., Mitsou V. (2009). Entropy, Orbits, and Spectra of Graphs. Analysis of Complex Networks: From Biology to Linguistics.

[16] Bollobàs B. (1998). Modern Graph Theory.

[17] Park J., Newman M.E.J. (2004). Statistical mechanics of networks. Phys. Rev. E Stat. Nonlinear Soft Matter Phys..

[18] Rényi A. On measures of entropy and information. Proceedings of the Fourth Berkeley Symposium on Mathematical Statistics and Probability.

[19] Leskovec J., Krevl A. SNAP Datasets: Stanford Large Network Dataset Collection. http://snap.stanford.edu/data.

[20] Clauset A., Tucker E., Sainz M. The Colorado Index of Complex Networks. http://icon.colorado.edu/.

[21] Burnham K.P., Anderson D.R. (2004). Multimodel inference: Understanding AIC and BIC in model selection. Sociol. Methods Res..

[22] Shang Y. (2016). Bounding extremal degrees of edge-independent random graphs using relative entropy. Entropy.

[23] Barabási A.L. (2016). Network Science.

[24] Estrada E., Rodríguez-Velázquez J.A. (2005). Subgraph centrality in complex networks. Phys. Rev. E.

[25] Shang Y. (2015). Laplacian Estrada and normalized Laplacian Estrada indices of evolving graphs. PLoS ONE.

[26] Ritchie M., Berthouze L., Kiss I.Z. (2017). Generation and analysis of networks with a prescribed degree sequence and subgraph family: Higher-order structure matters. J. Complex Netw..

[27] Shang Y. (2016). Localized recovery of complex networks against failure. Sci. Rep..

[28] Shang Y. (2016). On the likelihood of forests. Phys. A Stat. Mech. Its Appl..

[29] Jizba P., Ma Y., Hayes A., Dunningham J.A. (2016). One-parameter class of uncertainty relations based on entropy power. Phys. Rev. E.

[30] Bobkov S.G., Marsiglietti A. (2017). Variants of the Entropy Power Inequality. IEEE Trans. Inf. Theory.

[31] Trugenberger C.A. (2015). Quantum gravity as an information network self-organization of a 4D universe. Phys. Rev. D Part. Fields Gravit. Cosmol..

[32] Verlinde E.P. (2016). Emergent Gravity and the Dark Universe. arXiv.

